# Reconsidering the distribution of gray wolves

**DOI:** 10.24272/j.issn.2095-8137.2017.021

**Published:** 2017-05-18

**Authors:** Greger Larson

**Affiliations:** Palaeogenomics&Bio-Archaeology Research Network, Research Laboratory for Archaeology and History of Art, University of Oxford, UK

When attempting to understand where domestic plants and animals were domesticated, it is essential to consider the geographic distribution of the wild ancestor. Many domestic taxa now inhabit just about every continent thanks to their human-mediated dispersal which began soon after they were incorporated into the human niche. But just because sheep are now crucial to the economy of New Zealand and Wales, for example, does not mean that they were domesticated there. In fact, they could not have been since the wild ancestors of sheep were geographically restricted to a relatively small portion of Western Eurasia (Pedrosa et al., 2005).

Similarly, chickens, rabbits and camels are now found across the planet. Though wild populations of all three have also been moved by people and thrive in their new environments, it is only within the pre-historic natural ranges of the wild species that they could have been domesticated (Larson et al., 2014; Larson&Fuller, 2014; Wang et al., 2014).

The geographic origins of dogs have been contentious for several reasons, not the least of which is the widespread distribution of wolves across the Northern Hemisphere. The ability of wolves to colonise such a tremendous range from Portugal to Newfoundland means that, at least theoretically, dog domestication could have taken place anywhere (or more than once) across these longitudes.

Since 2002, multiple genetic studies of modern samples have suggested that dogs were domesticated in Southern East Asia (e.g., Wang et al., 2016a), though other studies have suggested alternative scenarios (e.g., Botigué et al., 2016; Frantz et al., 2016; Shannon et al., 2015). According to several canonical maps of wolf distribution, however, wolf populations never existed in this region. If true, then the conclusions based upon the genetic studies will have failed at the first hurdle since it would be impossible to domesticate a population that did not exist.

In order to establish the veracity of the commonly accepted maps, and to establish whether wolves were ever present in China, a new study conducted by Wang et al. (2016b) systematically searched for evidence for the presence of wolves. They began with a comprehensive literature search, but not content to rely on the testimony of others, they also visited three natural history museums and obtained 26 skins collected across China. Lastly, they identified 25 archaeological sites including wolf remains.

Taken in isolation, these individual lines of evidence could be questioned. The weight of all three together, however, suggests that at least historically, and most likely in pre-history as well, grey wolves maintained populations across China. An email exchange with the authors of the primary source that claimed wolves were absent from most of China revealed that the southern borders of the wolf distribution map were a great deal more equivocal than the boundary led readers to believe.

This result demonstrates the pitfalls of taking species distribution maps at face value. In this case, the line demarking the southern boundary of the grey wolf distribution has enormous ramifications. If wolves were present in central and southern China as recently as the second half of the 20th century, they were likely present in the preceding millennia and thus, they could have been the source of a domestication process in East Asia. This is not to say that dogs were definitively domesticated in China, but this result does at least remove a major hurdle that had been undermining that contention.

More generally, Wang et al. (2016b) demonstrates the power of a comprehensive due diligence to clarify what had been a long-standing, though ultimately insubstantial claim. In addition, this approach is key for ground-truthing and illuminating western scientists about the literature and records that have historically been difficult to penetrate. A great deal more information is sitting just under the surface and with collaborations between Eastern and Western scientist, the entire scientific community will benefit enormously, and answers to long-standing questions will be forthcoming. 
